# The effect of vitamin K4 supplementation on insulin resistance in individuals with type 2 diabetes: a double-blind randomised placebo-controlled clinical trial

**DOI:** 10.1007/s00394-023-03215-8

**Published:** 2023-08-08

**Authors:** Amani M. Ali, Maggie M. Abbassi, Nirmeen A. Sabry, May Fawzi, Shrook Mousa

**Affiliations:** 1 Department of Clinical Pharmacy, Faculty of Pharmacy, Cairo University, Cairo, Egypt; 2 Department of Internal Medicine, Kasr Alainy Faculty of Medicine, Cairo University, Cairo, Egypt

**Keywords:** Vitamin K, Diabetes mellitus, Type 2 diabetes, Insulin resistance, Lipid profile

## Abstract

**Purpose:**

The purpose of this study was to assess the possible clinical effects of vitamin K4 supplementation in individuals with type 2 diabetes namely insulin resistance, glycaemic control, and lipid profile.

**Methods:**

This was a prospective randomised double-blind placebo-controlled clinical trial. A total of 106 patients were randomised to receive either 1 mg of vitamin K4 (menadiol diacetate) or placebo for 24 weeks.

**Results:**

Ninety patients (n = 45 in each study group) were included in the final analysis. After 24 weeks, homeostatic model assessment of insulin resistance (HOMA-IR) (16.54 ± 7.81 vs. 29.09 ± 36.56, *P* = 0.027) and fasting serum insulin (FSI) (6.86 ± 3.45 vs. 11.13 ± 12.66 µU/ml, *P* = 0.032) were significantly lower in the vitamin K group compared to placebo. Additionally, triglycerides (TG) (144.94 ± 50.7 vs. 172.8 ± 101.5 mg/dl, *P* = 0.031) and very low-density lipoproteins (VLDL) levels (28.9 ± 9.88 vs. 34.6 ± 20.30 mg/dl, *P* = 0.027) decreased significantly in the vitamin K group after 24 weeks compared to baseline. Moreover, more patients in the vitamin K group (35.6%) had their antidiabetic medication doses reduced after 24 weeks compared to placebo (13.3%, *P* = 0.029).

**Conclusion:**

Vitamin K4 supplementation for 24 weeks is capable of improving insulin resistance and TG levels in individuals with type 2 diabetes. In addition, the improvement in insulin resistance was reflected in the decrease in antidiabetic medication doses. However, it did not affect fasting plasma glucose (FPG) or glycated haemoglobin (HbA_1_c).

**Trial registration:**

The study was registered on clinicaltrials.gov with ID: NCT04285450.

## Introduction

Diabetes is a common health problem that represents a major challenge to many healthcare systems [1]. Being the most prevalent endocrine disorder, it is estimated to affect 578 million people worldwide by 2030 [2]. Type 2 diabetes is the most widely occurring category that is characterised by defective insulin secretion and/or action [3]. Many factors are supposed to be involved in the pathophysiology of insulin resistance, among which, is vitamin K deficiency [4].

The term “vitamin K” is used to refer to a group of compounds that are primarily involved in the blood coagulation cascade [5]. Some of these are naturally occurring lipid-soluble entities like vitamin K1 and vitamin K2 [6]. Others are synthesised water-soluble compounds like vitamin K3 and vitamin K4 that are eventually converted to vitamin K2 in animal bodies [7]. In addition to its well-studied role in blood clotting, vitamin K has lately captured great attention out of having many other favourable functions in the human body [8]. Recent evidence suggests that higher vitamin K intake may be correlated with lower risk of coronary heart disease (CHD) and a lower risk of bone fractures [9, 10]. Moreover, vitamin K is supposed to have anticancer properties on account of opposing inflammation [11].

In diabetes, vitamin K intake is hypothesised to have beneficial effects in reducing the risk of type 2 diabetes development [12]. Also, it is reported to help in improving glycaemic control and insulin resistance in individuals with type 2 diabetes [13]. In addition, it may have a beneficial effect on the lipid profile of diabetics where higher vitamin K intake was found to correlate positively with high-density lipoprotein (HDL) [14]. Lastly, vitamin K is assumed to reduce the risk of cardiovascular disease (CVD)-related complications that are commonly found in type 2 diabetics [15].

Only a few studies assessed the effect of vitamin K supplementation on insulin resistance and glycaemic control [13–15]. These studies exclusively used vitamin K1 [16–19] and K2 [20, 21] as supplementation forms and both sides came up with conflicting results. In addition, none of them considered the use of synthetic forms like vitamin K3 and K4. Vitamin K4, menadiol diacetate, is a hydrophilic synthetic form of vitamin K that is metabolised inside the body into vitamin K3, menadione. Menadione has three main metabolic pathways. It is either alkylated in the liver to menaquinone-4 (vitamin K2), or absorbed to lymphatics or conjugated in order to be excreted in the bile and urine [22]. Vitamin K4 is available in oral form in Egypt and has the advantage of better absorption in the gastrointestinal tract being a water-soluble vitamer compared to phylloquinone and menaquinones which are lipid-soluble derivatives [22]. Therefore, the current study aimed to further scrutinise the effect of vitamin K supplementation in the form of vitamin K4 on insulin resistance, glycaemic control, and lipid profile of individuals with type 2 diabetes.

## Methods

### Trial design, randomization and intervention

This was a randomised interventional double-blind placebo-controlled clinical trial. Patients were recruited from the Kasr Alainy outpatient diabetes and endocrinology clinic in the period from October 2019 to March 2022.

Two sets of random numbers were generated using the website www.randomizer.org. Patients were then randomly allocated to one of the two study arms. The first group was dispensed 1 mg of menadiol diacetate (vitamin K4) that was obtained from K-VITON^®^ 10 mg sugar-coated tablets produced by Kahira Pharmaceuticals and Chemical Industries Company, Cairo, Egypt. This is the only available concentration in the Egyptian market, therefore, tablets were crushed and one-tenth of each by weight was placed in a hard gelatine capsule. The placebo group was dispensed lactose 30 mg obtained from Alamia Company for Chemicals, Cairo, Egypt. Lactose was packed in similar hard gelatine capsules. Capsules were secured from Hochster Pharmaceuticals Company, Cairo, Egypt. Since this was a pilot batch, not an industrial one, capsules were filled in the laboratory and weighed before and after filling. Patients were advised to take one capsule daily after their largest meal. Patients were followed up for 24 consecutive weeks.

### Participants

Individuals with type 2 diabetes were considered eligible for joining the study if their age was ranging from 18 to 65 years old, with alanine aminotransferase (ALT) levels up to 33 U/L for males and up to 25 U/L for females, albumin levels ≥ 3.5 g/dL, bilirubin up to 1.1 mg/dL [23] and normal international normalized ratio (INR) and serum creatinine levels.

Exclusion criteria included individuals with type 1 diabetes, use of vitamin supplements except for vitamin B complex, smoking, patients requiring anti-coagulant therapy including patients with prosthetic valves, deep vein thrombosis (DVT), pulmonary embolism (PE), atrial fibrillation, and valvular heart disease. Moreover, patients with previous thromboembolic events, myocardial infarction (MI), stroke, and embolization were excluded. Patients who were pregnant, breastfeeding, on hormonal therapy, contraceptive pills, glucocorticoids, thiazide diuretics, atypical antipsychotics, cholestyramine, antibiotics, coumarins, and lipid lowering agents in addition to patients who suffer from intestinal malabsorption syndrome, cholestasis or steatorrhea were excluded as well.

### Dietary assessment

All patients were educated about vitamin K rich food (containing more than 50 μg/100 g of phylloquinone or dihydrophylloquinone or more than 5 μg/100 g of menaquinone-4 according to the United States Department of Agriculture and Agricultural Research Service (USDA) [24] that are available in Egypt. Patients were advised to keep the dietary consumption of these foodstuffs constant all over the study and were followed up every four weeks to ensure that there is no change in the intake over the study period and to detect any difference if any between the two study groups. Additionally, a specially designed picture-based form was given out to patients where they were requested to report the number of times per day they consumed any of these foods.

The average daily vitamin K intake of the study participants was calculated based on the number of portions of vitamin K rich food, as mentioned earlier, multiplied by the vitamin K content of these foodstuffs as per the USDA.

### Follow-up and compliance

At baseline, 12 weeks and 24 weeks, patients were asked to come to the clinic after an overnight fast of 10 h to be tested for their FPG and FSI besides a full lipid profile. Then, they were given 75 gm of anhydrous glucose for the oral glucose tolerance test (OGTT). They also had their HbA_1_c, vitamin K, and INR levels besides change in antidiabetic medication doses and anthropometric measurements checked at the same timepoints. Anthropometric assessment included the measurement of the patients’ height, weight, waist circumference, and hip circumference for the subsequent calculation of body mass index (BMI) and waist-to-hip ratio (WHR). The blinded physician had the authority to intensify or decrease the doses of antidiabetic medications based on patient symptoms besides FPG and HbA_1_c readings. Renal and hepatic function checks were done at baseline and after 24 weeks. Every 4 weeks, the remaining capsules in the patient’s container were counted to assess for compliance.

### Study outcomes

The primary outcome for the study was insulin resistance assessed by HOMA-IR. Secondary outcomes included: FPG and 2 h postprandial plasma glucose (2 h-PP PG), FSI, HbA_1_c, lipid profile, body mass index (BMI), waist-to-hip ratio (WHR), and vitamin K levels.

### Sample size calculation

Sample size calculation was performed using G*Power software version 3.1.9.2, Germany. Relying on literature effect size of 0.617 in HOMA-IR as a primary outcome [25], 86 patients were required to achieve 80% power at 5% level of significance. Assuming a dropout rate of 10%, then a total sample size of 96 would be required which means 48 patients in each group.

### Statistical analysis

The Statistical Package for the Social Sciences (SPSS) software package version 22 was used for all analyses (SPSS Inc., Chicago, IL, USA). Baseline and 24-weeks characteristics were represented as mean ± SD for continuous variables and frequencies (percentage) for nominal variables. The final analysis was based on a modified intention to treat (ITT) analysis where patients who have attended the middle follow-up appointment in the 12th week were considered in the final analysis. Missing data were predicted by multiple imputation (MI) based on the average of five iterations.

Paired and unpaired *t* test were used for the comparison of continuous variables in the same group and between different groups respectively. Chi-square test was used to compare the distribution of categorical variables among different groups. Two-sided P values < 0.05 were considered statistically significant. Graphs were prepared using GraphPad Prism Software version 9.5.1.

## Results

**Fig. 1:**
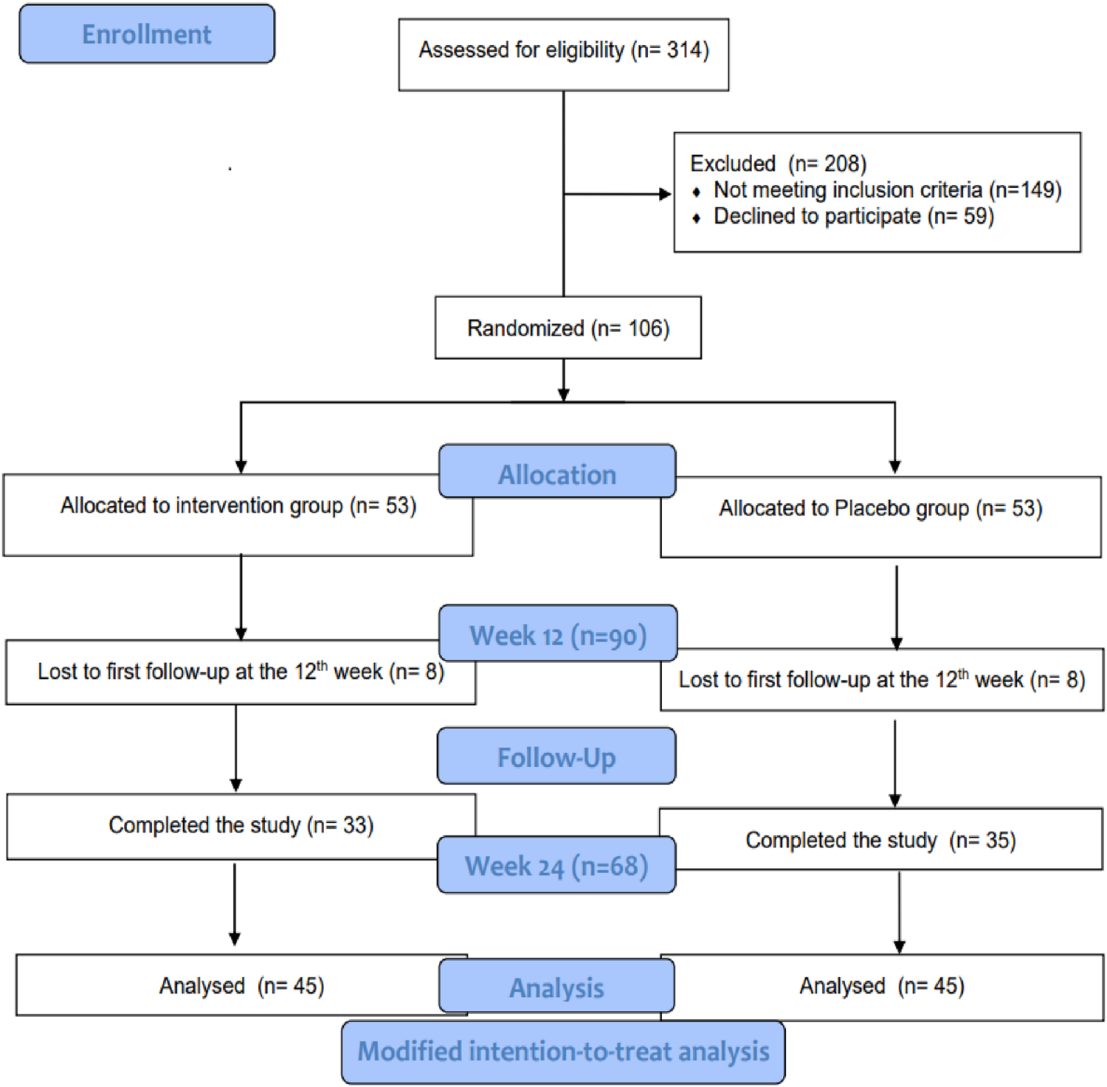
CONSORT flowchart for the study participants

In the current study, a total of 90 patients reached the middle of the follow-up period after 12 weeks and hence, were included in the final analysis on a modified ITT basis (Fig. 1). At baseline, there was no significant difference between the two study groups in any of the demographic or biochemical parameters except for the INR which was significantly lower in the intervention group at baseline (Table 1). However, all study participants had normal INR values that ranged from 1.18 to 0.85. Around 89% of the study participants were females. The mean age was 53.11 ± 8.27 years in the placebo group vs. 49.68 ± 8.26 years in the intervention group. The antidiabetic treatment of the study population included insulin and metformin for the majority of patients (Table 1). Insulin resistance of the study population as assessed by HOMA-IR was 8.39 ± 8.58 in the placebo group vs. 12.93 ± 31.06 in the intervention group at baseline.

**Table 1:** Demographic data and baseline parameters of the study participants

Parameter	Baseline parameters		
	Placebo (n = 45)	Vitamin K (n = 45)	P value ^a^
Age (years)	53.11 ± 8.27	49.68 ± 8.26	0.053
Female, n (%)	40 (88.9%)	40 (88.9%)	1.000^b^
Antidiabetic treatment n (%)			
OADs only	15 (33.3%)	18 (40%)	0.781^b^
Insulin only	4 (8.9%)	3 (6.7%)	
Insulin + metformin	26 (57.8%)	24 (53.3%)	
Hypertension, n (%)	18 (40%)	19 (42.2%)	1.000^b^
Weight (kg)	90.12 ± 17.32	87.26 ± 14.4	0.397
BMI (kg/m^2^)	35.59 ± 6.01	34.6 ± 5.33	0.411
Waist circumference (cm)	114.04 ± 10.98	110.84 ± 10.75	0.166
WHR	0.934 ± 0.0602	0.926 ± 0.057	0.534
FPG (mg/dl)	169.8 ± 60.28	164.82 ± 74.19	0.723
2-h PP PG (mg/dl)	302.6 ± 71.8	303.5 ± 92.14	0.958
FPI (µU/ml)	19.97 ± 15.8	32.8 ± 81.29	0.301
HOMA-IR	8.39 ± 8.58	12.93 ± 31.06	0.347
HbA_1_c (%)	8.37 ± 1.66	8.37 ± 1.63	0.995
Vitamin K1 level (pg/ml)	131.4 ± 83.4	122.7 ± 82.48	0.623
Total cholesterol (mg/dl)	198.3 ± 39.9	205.9 ± 30.25	0.308
Triglycerides (mg/dl)	155.8 ± 85.07	172.8 ± 101.5	0.392
HDL (mg/dl)	44.3 ± 11.66	44.04 ± 8.35	0.901
LDL (mg/dl)	125.74 ± 34.08	129.4 ± 27.3	0.574
VLDL (mg/dl)	31.2 ± 17.1	34.6 ± 20.30	0.390
TC:HDL ratio	4.67 ± 1.27	4.85 ± 1.32	0.503
INR	1.002 ± 0.089	0.96 ± 0.066	0.015*
eGFR (ml/min)	135.22 ± 50.72	138.83 ± 51.76	0.739

Data are represented as means ± SD for continuous data and number (percentage) for nominal data

*OADs* oral antidiabetic medications, *BMI* body mass index, *WHR* waist-to-hip ratio, *FPG* fasting plasma glucose, *2-h PP PG* two-hour post-prandial plasma glucose, *FPI* fasting plasma insulin, *HOMA-IR* homeostatic medal assessment for insulin resistance, *HbA*_*1*_*c* glycated haemoglobin, *HDL* high-density lipoprotein, *LDL* low-density lipoprotein, *VLDL* very low-density lipoprotein, *TC:HDL-C ratio* total cholesterol to high-density lipoprotein cholesterol ratio, *INR* international normalized ratio, *eGFR* estimated glomerular filtration rate

^*^P values < 0.05 are considered statistically significant

^a^Independent samples *t* test

^b^Chi-square test

Studying the change from baseline in the study outcomes at the study timepoints (Table 2) shows that HbA_1_c significantly decreased in the intervention group (– 0.2 ± 0.99% vs 0.2 ± 1.1% in the placebo group, *P* = 0.045) in addition to a significant increase in the HDL at the 12th week (4.04 ± 13.8 mg/dl vs – 1.3 ± 7.6 mg/dl in the placebo group, *P* = 0.026). However, these two effects were not sustained to the 24th week.

**Table 2:** Change in the study outcomes from baseline at 12 weeks and 24 weeks

Parameter	Change from baseline at 12 weeks		P value^a^	Change from baseline at 24 weeks		P value^a^
	Placebo (n = 45)	Vitamin K (n = 45)		Placebo (n = 45)	Vitamin K (n = 45)	
FPG (mg/dl)	2.67 ± 73.26	10.9 ± 76.9	0.604	– 4.4 ± 78.4	10.8 ± 78.3	0.355
2-h PP PG (mg/dl)	10.5 ± 81.54	3.9 ± 110.28	0.750	17.36 ± 82.2	7.5 ± 110.97	0.634
FPI (µU/ml)	0.69 ± 15.09	– 15.86 ± 80.9	0.181	9.11 ± 38.82	– 16.25 ± 80.82	0.061
HOMA-IR	0.1 ± 9.3	– 5.9 ± 30.5	0.204	2.73 ± 14.08	– 6.06 ± 30.81	0.085
HbA_1_c (%)	0.2 ± 1.1	– 0.2 ± 0.99	0.045*	0.18 ± 1.43	0.01 ± 1.26	0.541
Total cholesterol (mg/dl)	6.06 ± 41.02	7.13 ± 29.18	0.886	7.9 ± 47.17	– 0.66 ± 28.5	0.296
Triglycerides (mg/dl)	2.44 ± 66.9	– 4.8 ± 69.79	0.614	3.6 ± 110.4	– 27.85 ± 83.67	0.131
HDL (mg/dl)	– 1.3 ± 7.6	4.04 ± 13.8	0.026*	– 0.12 ± 11.55	– 0.66 ± 9.15	0.807
LDL (mg/dl)	3.9 ± 35.14	1.7 ± 27.21	0.734	0.39 ± 36.8	– 4.8 ± 27.13	0.447
VLDL (mg/dl)	0.44 ± 13.42	– 0.97 ± 13.93	0.623	– 0.38 ± 20.22	– 5.7 ± 16.74	0.178
TC:HDL ratio	0.24 ± 0.93	– 0.15 ± 1.19	0.088	0.18 ± 1.4	0.01 ± 1.59	0.597

Data are represented as means ± SD

*FPG* fasting plasma glucose, *2-h PP PG* two-hour post prandial plasma glucose, *FPI* fasting plasma insulin, *HOMA-IR* homeostatic medal assessment for insulin resistance, *HbA*_*1*_*c* glycated haemoglobin, *HDL* high-density lipoprotein, *LDL* low-density lipoprotein, *VLDL* very low-density lipoprotein, *TC:HDL-C ratio* total cholesterol to high-density lipoprotein cholesterol ratio

*P values < 0.05 are considered statistically significant

^a^Independent samples *t* test

After 24 weeks of vitamin K4 supplementation, the intervention group had significantly lower FSI (11.13 ± 12.66 µU/ml in the placebo group vs. 6.86 ± 3.45 µU/ml in the intervention group, *P* = 0.032) and HOMA-IR (29.09 ± 36.56 in the placebo group vs. 16.54 ± 7.81 in the intervention group, *P* = 0.027) than the placebo group. However, no significant difference was observed in terms of FPG, PP PG, and HbA_1_c (Table 3).

**Table 3:** Characteristics of the study participants at 24 weeks

Parameter	24-weeks parameters		
	Placebo (n = 45)	Vitamin K (n = 45)	P value ^a^
Weight (Kg)	90.5 ± 15.1	87.3 ± 13.6	0.308
BMI (kg/m^2^)	35.8 ± 5.49	34.47 ± 5.05	0.227
Waist circumference (cm)	112.6 ± 8.9	109.75 ± 8.45	0.114
WHR	0.921 ± 0.041	0.922 ± 0.049	0.954
FPG (mg/dl)	165.39 ± 55.5	175.67 ± 56.88	0.388
2-h PP PG (mg/dl)	319.98 ± 67.95	311.06 ± 78.16	0.565
FPI (µU/ml)	29.09 ± 36.56	16.54 ± 7.81	0.027*
HOMA-IR	11.13 ± 12.66	6.86 ± 3.45	0.032*
HbA_1_c (%)	8.56 ± 1.68	8.38 ± 1.5	0.600
Vit K1 level (pg/ml)	76.14 ± 87.39	118.28 ± 249.86	0.288
Vitamin K1 intake (µg/day)	158.03 ± 121.96	141.42 ± 13.31	0.534
Total cholesterol (mg/dl)	206.27 ± 32.58	205.28 ± 32.69	0.887
Triglycerides (mg/dl)	159.42 ± 109.27	144.94 ± 50.7	0.422
HDL (mg/dl)	44.18 ± 9.7	43.38 ± 7.8	0.668
LDL (mg/dl)	126.14 ± 31.78	124.61 ± 26.9	0.806
VLDL (mg/dl)	30.81 ± 19.68	28.9 ± 9.88	0.565
TC: HDL ratio	4.85 ± 1.164	4.86 ± 1.1	0.951
*Antidiabetic treatment change*			
Doses increased	14 (31.1%)	7 (15.6%)	0.029^b^*
Doses decreased	6 (13.3%)	16 (35.6%)	
No change	25 (55.6%)	22 (48.9%)	
Compliance (%)	95.86 ± 4.77	98.29 ± 2.19	0.003*
INR	0.97 ± 0.065	0.94 ± 0.073	0.071

*BMI* body mass index, *WHR* waist-to-hip ratio, *FPG* fasting plasma glucose, *2-h PP PG* two-hour post prandial plasma glucose, *FPI* fasting plasma insulin, *HOMA-IR* homeostatic medal assessment for insulin resistance, *HbA*_*1*_*c* glycated haemoglobin, *HDL* high-density lipoprotein, *LDL* low-density lipoprotein, *VLDL* very low-density lipoprotein, *INR* international normalized ratio

Data are represented as means ± SD for continuous data and number (percentage) for nominal data

^*^P values < 0.05 are considered statistically significant

^a^Independent samples *t* test

^b^Chi-square test

**Fig. 2:**
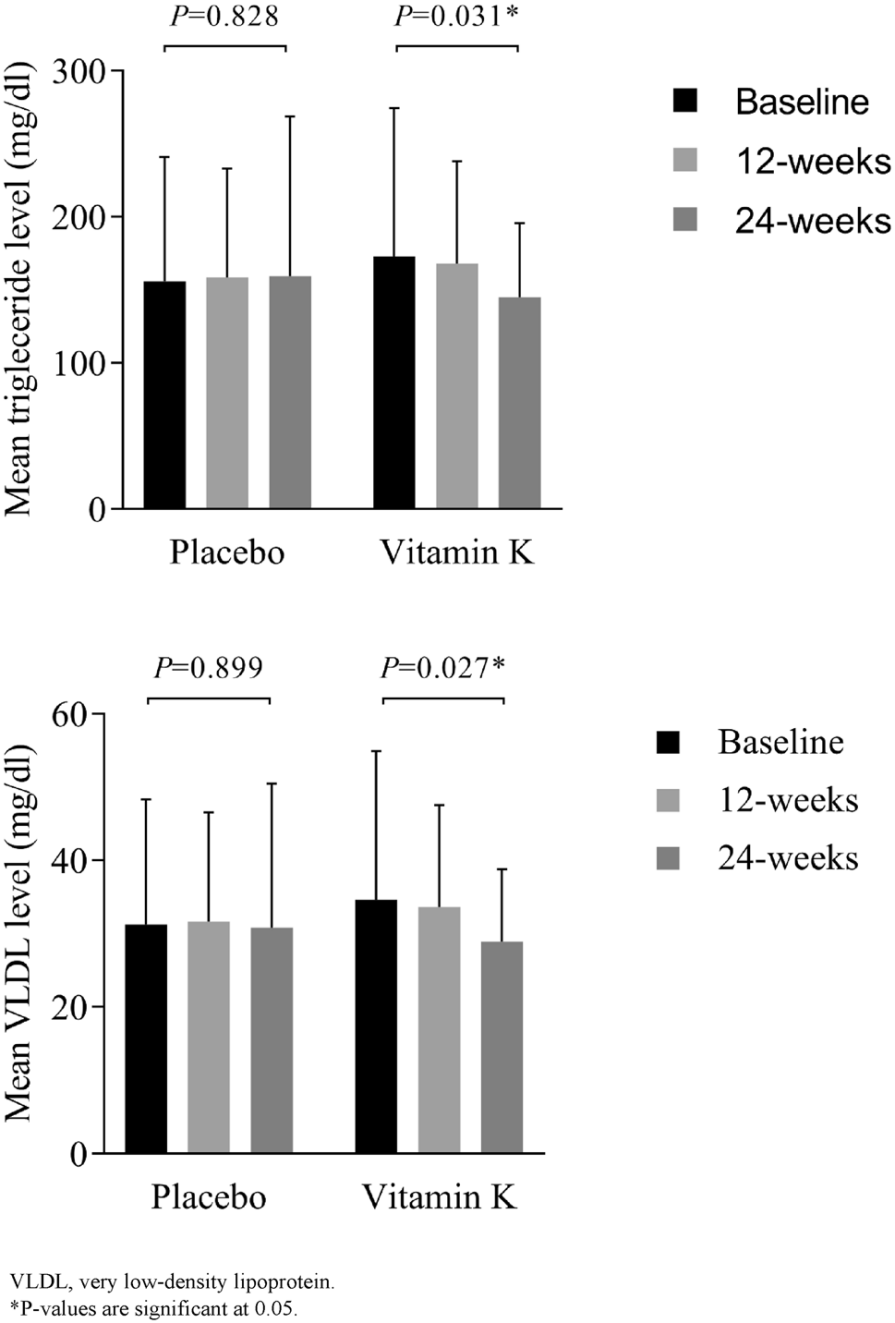
The effect of vitamin K4 supplementation on mean triglycerides and VLDL levels

Regarding the lipid profile of the patients, there was no significant difference between the two groups in terms of total cholesterol (TC), TG, low-density lipoprotein (LDL), VLDL, HDL or TC:HDL risk factor after 24 weeks. However, paired *t* test comparison showed a significant reduction in plasma TG and VLDL in the intervention group after 24 weeks compared to baseline (TG: 172.8 ± 101.5 mg/dl at baseline vs. 144.94 ± 50.7 mg/dl after 24 weeks, *P* = 0.031) (VLDL: 34.6 ± 20.30 mg/dl at baseline vs. 28.9 ± 9.88 mg/dl after 24 weeks, *P* = 0.027) (Fig. 2).

Measuring plasma vitamin K levels showed no significant difference between the two groups neither at baseline nor after 24 weeks. The average daily vitamin K1 intake of the study patients throughout the trial period was 158.03 ± 121.96 µg/day in the placebo group and 141.42 ± 13.31 µg/day in the intervention group, *P* = 0.534 (Table 3). The compliance was significantly higher in the intervention group, however, none of the study participants consumed less than 80% of the capsules. Also, none of the participants reported any adverse effects of the administered supplement or placebo capsules.

Antidiabetic treatment for the study population included metformin, glibenclamide besides injectable insulin. The majority of the patients in the current trial did not require any change in their antidiabetic medication regimens (55.6% in the placebo group and 48.9% in the intervention group). Nonetheless, 31.1% in the placebo group required treatment intensification vs. 15.6% in the intervention group, and 13.3% required dosage reductions in the placebo group vs. 35.6% in the intervention group (*P* = 0.029).

## Discussion

In the current study, we demonstrated that vitamin K4 supplementation for 24 weeks was capable of reducing FSI and insulin resistance assessed by HOMA-IR with no significant effects on FPG or 2-h PP PG levels or HbA_1_c. It was also shown to significantly reduce serum TG and VLDL without changing TC, HDL or LDL. In addition, there was a significant difference between the two groups in the change of the doses of their antidiabetic medications.

Vitamin K3, which is formed following vitamin K4 intake, is assessed to be more potent than natural vitamin Ks and serves as a precursor to vitamin K2 inside the body [26, 27]. Vitamin K acts as an important catalyst for ɣ-glutamyl carboxylase enzyme. This enzyme turns on a group of plasma proteins, called ɣ-carboxyglutamic acid (Gla)-proteins, through their carboxylation. Osteocalcin (OC) is one of the most crucial vitamin K-dependent proteins that are involved in glucose metabolism and homeostasis [28]. It has been previously hypothesised that there is a continuous signalling cycle between pancreatic β-cells and the skeletal system where insulin binding to its receptor on osteoblasts induces osteocalcin release that in consequence regulates pancreatic insulin production and improves insulin resistance [29].

Studying the effect of vitamin K4 on glycaemic control of individuals with type 2 diabetes in the current study revealed a significant decrease in the fasting serum insulin and HOMA-IR in the intervention group compared to the placebo group after 24 weeks. Most interventional studies that used vitamin K1 as a supplementation form reported no effect on insulin resistance. The study by Rasekhi et al. reported a significant reduction in postprandial glucose and postprandial insulin and significant increase in insulin sensitivity index with no effect on FSI or insulin resistance upon 4 weeks of vitamin K1 supplementation [16]. These contrasting findings to ours may be attributed to the fact that the population of this study was women who were premenopausal and prediabetic with a mean age of 40 years while most of our study participants were women with type 2 diabetes with a mean age of around 50 years. As obvious, age is well-proven to be associated with progressive glucose intolerance, insulin resistance, and decreased insulin secretion [30].

Likewise, another thesis illustrated that the administration of 500 µg vitamin K1 daily for 3 weeks did not affect HOMA-IR [18]. Similarly, Kumar et al., reached the same conclusion although they considered a higher dose, 1 mg, for a longer period, 12 months [19]. Yoshida et al. [17] agreed in part with these results where they reported no significant change in HOMA-IR in women who participated in their study that involved 500 µg/day of vitamin K1 supplementation for 36 months. However, they observed a significant reduction in HOMA-IR and FSI in male participants. The authors attributed this observation to the circumstance that women in their study had higher BMI than enrolled men and this affected their response to vitamin K supplementation.

In contrast, studies that adopted vitamin K2 as a supplementation form showed more promising results in improving insulin resistance. Two recent studies [20, 21] used menaquinone-7 for 12 weeks in 2 different doses, 200 µg and 360 µg daily, reported a significant reduction in the vitamin K group’s HOMA-IR and FSI after 12 weeks compared to baseline without significant intergroup variation. This agrees with our findings even though this was observed after 24 weeks of K4 supplementation. Also, both studies revealed a significant reduction in FPG and HbA_1_c in the intervention group compared to placebo. It is worth noting that these two studies were the only supplementation studies that considered people with type 2 diabetes as a study population. In agreement to these findings, in our study, there was a transient decrease in the HbA_1_c of the intervention group in the 12th week that did not keep up till the 24th week. Comparing the baseline characteristics of our study participants with those of the aforementioned trials reveals that ours are much more insulin resistant (denoted by higher serum levels of insulin and higher HOMA-IR values at baseline). Obviously, this can be linked to the higher weight and BMI of our study population at baseline since obesity has been extensively regarded as a direct cause of β-cell damage and impaired insulin sensitivity [31, 32]. Moreover, the observed improvement in HbA_1_c at the 12th week in the vitamin K group (Table 2) with subsequent reduction in antidiabetic medication doses might have contributed to the lack of effect on blood glucose levels by the end of the trial.

In the present study, vitamin K4 supplementation was shown to significantly reduce serum TG and VLDL in individuals with type 2 diabetes with no significant effects on serum cholesterol. Similarly, another study investigated the effect of 8-week vitamin K1 supplementation in female patients with rheumatoid arthritis concluded that it has no effect on any of the lipid profile markers [33]. Similar conclusion was reached by the two randomised controlled trials that examined menaquinone-7 in type 2 diabetes [21, 34]. Conversely, the study by Kristensen et al., [35] showed that K1 supplementation for 6 weeks in postmenopausal women did increase serum triglycerides in addition to decreasing HDL-C.

For a long time, insulin resistance and high insulin levels have been linked to increased levels of VLDL and triglycerides [36]. In addition, TG levels and TG:HDL ratio have been considered possible indicators for insulin resistance with better predictability in Caucasians compared to African Americans [37, 38]. Increased level of triglycerides in insulin resistant individuals is interpreted by high production of hepatic triglycerides together with reduced clearance from circulation [39]. In the current study population, although non-significant, the intervention group had a higher triglyceride and VLDL levels at baseline than the placebo group. This explains the non-significant difference observed between the two groups in the same variables after 24 weeks despite the significant decrease observed in the intervention group upon comparing 24-weeks to baseline data. This drop in plasma TG in the vitamin K4 group can be clarified by the decrease in fasting serum insulin and the improvement of insulin resistance as assessed by HOMA-IR by around 50% in the same group.

Although, there is a debate about the sufficient daily intake of vitamin K, our study population seemed to consume sufficient amounts of phylloquinone which constitutes around 90% of vitamin K dietary intake [40]. Also, there is no general agreement neither on a specific biomarker to reflect vitamin K levels in the body, nor on a specific level of serum vitamin K to be considered sufficient or deficient [41]. Even though vitamin K4 is proven to be metabolised in the body to vitamin K3 [42], there is no evidence that this would be reflected in plasma phylloquinone levels. Contrariwise, phylloquinone is proven to be converted to vitamin K3 in vivo [43]. This might explain the lack of increase in phylloquinone levels in the supplemented group.

The current trial has many points of strength. These may include a relatively long follow-up period in addition to reporting the change in patients treatment across the follow-up period. Given that this was a single-centred trial, this ensured similar dietary vitamin K consumption among the study participants across the follow-up period because their socioeconomic level was very close. Furthermore, this study is a valuable addition to the current evidence of the role of vitamin K in diabetes since patients were supplemented with a vitamin K form that is investigated for the first time.

One limitation of the study is that vitamin K-dependent proteins like osteocalcin were not assessed. Also, the drop-out rate was relatively high which was attributed to the interruption of the study period by COVID-19 lockdown. Moreover, the assessment of dietary vitamin K intake depended mainly on a picture-based form of vitamin K rich food besides the lack of assessment of caloric, fibres, and other nutrients intake. Yet, picture-based assessment was the most feasible method as there were some illiterate subjects among the study participants. Besides, the compliance of patients to their antidiabetic medication was not contemplated. Also, patients were not educated nor followed-up for changes in their physical activity.

In conclusion, 1 mg vitamin K4 supplementation might help in the reduction of insulin resistance in individuals with type 2 diabetes which could help in the reduction of the required antidiabetic treatment doses. Besides, it may have a role in the reduction of serum TG and VLDL. More controlled studies with higher doses of supplementation and longer duration would be important to clarify if there is possible effect on blood glucose levels and long-term glycaemic control as measured by HbA_1_c.

## Data Availability

Not available.
